# Secondary Autochthonous Outbreak of Chikungunya, Southern Italy, 2017

**DOI:** 10.3201/eid2511.180949

**Published:** 2019-11

**Authors:** Flavia Riccardo, Giulietta Venturi, Marco Di Luca, Martina Del Manso, Francesco Severini, Xanthi Andrianou, Claudia Fortuna, Maria Elena Remoli, Eleonora Benedetti, Maria Grazia Caporali, Francesca Fratto, Anna Domenica Mignuoli, Liliana Rizzo, Giuseppe De Vito, Vincenzo De Giorgio, Lorenzo Surace, Francesco Vairo, Paola Angelini, Maria Carla Re, Antonello Amendola, Cristiano Fiorentini, Giulia Marsili, Luciano Toma, Daniela Boccolini, Roberto Romi, Patrizio Pezzotti, Giovanni Rezza, Caterina Rizzo

**Affiliations:** Istituto Superiore di Sanità, Rome, Italy (F. Riccardo, G. Venturi, M. Di Luca, M. Del Manso, F. Severini, X. Andrianou, C. Fortuna, M.E. Remoli, E. Benedetti, M.G. Caporali, A. Amendola, C. Fiorentini, G. Marsili, L. Toma, D. Boccolini, R. Romi, P. Pezzotti, G. Rezza);; European Centre for Disease Prevention and Control, Stockholm, Sweden (X. Andrianou);; Regione Calabria, Calabria, Italy (F. Fratto, A.D. Mignuoli, L. Rizzo);; ASP di Catanzaro, Calabria (G. De Vito, V. De Giorgio, L. Surace);; National Institute for Infectious Diseases, Rome (F. Vairo);; Emilia-Romagna Region, Bologna, Italy (P. Angelini);; University of Bologna, Bologna (M.C. Re);; Ospedale Pediatrico Bambino Gesù, Rome (C. Rizzo)

**Keywords:** Chikungunya, outbreak, autochthonous, viruses, CHIKV, chikungunya virus, Calabria, Italy

## Abstract

In 2017, a chikungunya outbreak in central Italy later evolved into a secondary cluster in southern Italy, providing evidence of disease emergence in new areas. Officials have taken action to raise awareness among clinicians and the general population, increase timely case detection, reduce mosquito breeding sites, and promote mosquito bite prevention.

In 2007 (*1*) and 2017 (*2*), local outbreaks of human chikungunya infection occurred in Italy; both outbreaks were caused by the East/Central/South African strain of chikungunya virus (CHIKV). Both outbreaks were sustained by the invasive mosquito *Aedes albopictus*, largely established in Italy and other countries in southern Europe (*3*). In 2017, France and Italy reported local transmission of CHIKV (*2**,**4*). However, in France, the number of cases was limited; whereas in Italy, 499 probable and confirmed cases of locally acquired CHIKV infection occurred, of which 270 were laboratory confirmed as per the European Union (EU) case definition of June 22, 2018 (https://eur-lex.europa.eu/legal-content/EN/TXT/PDF/?uri=CELEX:32018D0945&from=EN#page=13).

 After local CHIKV transmission was confirmed in the seaside city of Anzio (Lazio region, central Italy) in 2017, the outbreak spread within the region, including in the city of Rome (*5**–**7*). The beginning of the outbreak was traced back to June 2017 (Figure 1). Subsequently, an outbreak developed in Guardavalle Marina, a small village of 2,346 inhabitants in the Calabria region of southern Italy, causing 100 probable/confirmed cases (Figures 1, 2).

**Figure 1:**
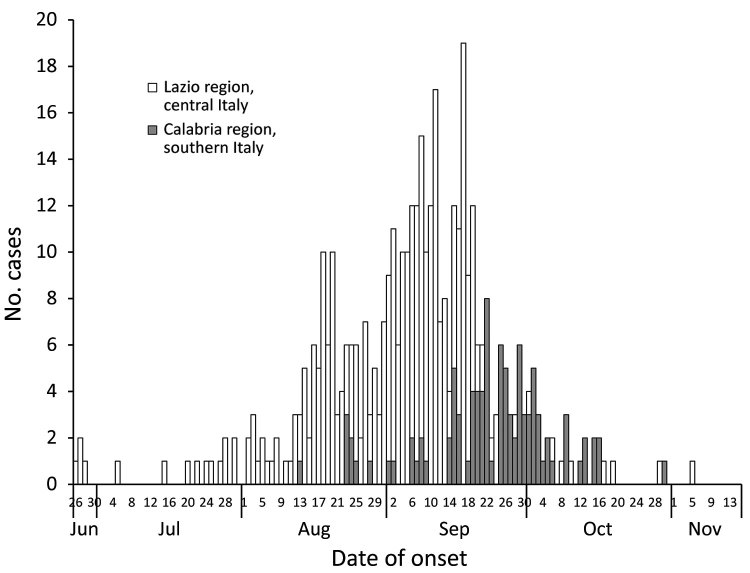
Epidemic curve for 499 cases of chikungunya (probable and confirmed) in central and southern Italy, June 26–November 15, 2017.

**Figure 2:**
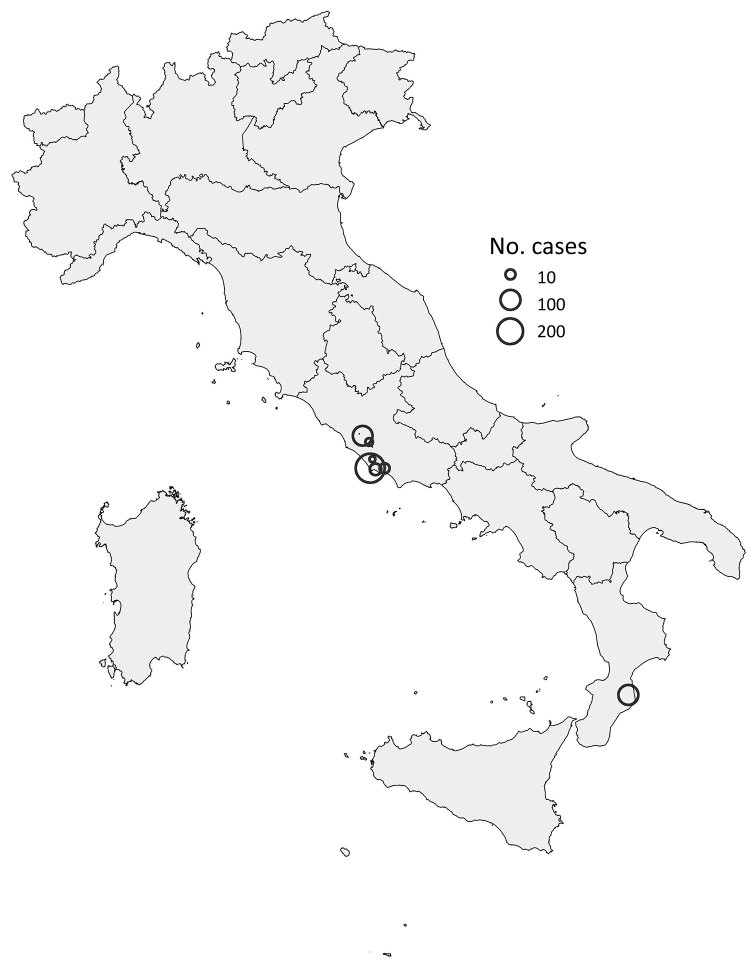
Location and size of clusters of 499 cases of chikungunya (probable and confirmed), by municipality, in central and southern Italy, June 26–November 15, 2017.

## The Cases

In August 2017, a patient from Anzio arrived in Guardavalle Marina the day before onset of symptoms that met the clinical criteria of the EU case definition for chikungunya (suspected case); subsequently, more cases in the village were reported. For most case-patients, the clinical course of the disease was fairly mild. All patients with confirmed/probable cases reported fever, and 99% reported severe and persistent joint pain, which seemed to be the most indicative symptom of chikungunya (Appendix Table 2). Phylogenetic analysis of isolates from patients with confirmed cases and from mosquito pools showed that the virus strains from Lazio and Calabria were similar to the East/Central/South African strains detected in Pakistan and India (*2**,**8*). Neither strain contained the A226V mutation that was detected in the strain responsible for the 2007 outbreak in Italy (*1*).

The epidemiologic and microbiological evidence (Appendix) supports the hypothesis that the Guardavalle Marina outbreak originated from Lazio rather than from an independent introduction. The overall clinically observed attack rate in Guardavalle Marina was 4.3%, similar to the 5.4% rate reported during the 2007 outbreak in Castiglione di Cervia but much lower than the 34% rate reported in La Réunion (*9*) or the high attack rates typically reported by other affected tropical countries (*10*). The duration of the Guardavalle Marina outbreak (2 months) was also closer to the duration of the outbreak in Castiglione di Cervia (July–September 2007) than to the duration of the outbreak in La Réunion (March 2005–April 2006) (*11*). 

Clinically observed attack rates progressively increased with patient age (Appendix Table 1). This pattern was also observed during the 2007 outbreak in Italy (*1*) and was mainly attributed to older age being a proxy for specific behavior linked to higher exposure to bites from *Ae. albopictus* mosquitoes (*12*). We cannot exclude underestimation of the observed attack rate in Guardavalle Marina, even though extensive door-to-door case finding was feasible and performed, given the small size of the village.

Notwithstanding the lack of the A226V mutation, the 2017 strain was introduced and rapidly spread with evidence of disease emergence in new areas. Statistically significant case clustering was confirmed by spatiotemporal data analysis in Guardavalle Marina (Appendix Figure 2), and *Ae. albopictus* mosquito vector competence for the 2017 strain was recently found to be comparable to competence for the 2007 mutated strain (*13*).

Delayed clinical detection of cases, possibly resulting from lack of CHIKV infection awareness among clinicians (*14*), and hence delayed testing for laboratory confirmation, could explain the size and extension of the 2017 outbreak. The fact that the outbreak was contained in a relatively short time after detection could be the result of the combination of targeted vector control interventions (Appendix), cooling temperatures, and increased rainfall during October–November 2017.

## Conclusions

Since 2011, Italy has had a yearly updated plan for the surveillance and control of human infections caused by CHIKV; the plan includes designated reference laboratories, vector control, and blood/transplant safety measures (Appendix). After the 2017 outbreak, a “presumed local cluster” was defined as occurring when local transmission of CHIKV is suspected for >2 cases, of which only 1 case needs to be laboratory confirmed. It is sufficient for the second case to be suspected on clinical and epidemiologic grounds pending laboratory confirmation. This new definition, aimed at triggering more timely blood/transplant safety and outbreak response measures, was successfully implemented in 2018. Actions to raise awareness among clinicians and the general population are being designed as part of a joint effort of animal and human public health institutions and universities coordinated by the Italy Ministry of Health. These actions are aimed at increasing timely case detection, reducing the number of mosquito breeding sites, and promoting individual prevention of mosquito bites.

Appendix Additional methods and results for study of secondary autochthonous outbreak of chikungunya, southern Italy, 2017.
