# Supplementary material for: Secondary Autochthonous Outbreak of Chikungunya, Southern Italy, 2017
Source: Emerg Infect Dis. 2019 Nov;25(11):2093–5. doi: 10.3201/eid2511.180949 (PMC6810187; doi:10.3201/eid2511.180949)
Supplement: Appendix — Additional methods and results for study of secondary autochthonous outbreak of chikungunya, southern Italy, 2017. [file 18-0949-Techapp-s1.pdf]

Article DOI: https://doi.org/10.3201/eid2511.180949

Secondary Autochthonous Outbreak of Chikungunya, Southern Italy, 2017

Appendix

Outbreak investigation in Guardavalle Marina

Surveillance of Chikungunya virus (CHIKV) infections in Italy is part of an integrated national surveillance and response plan (*1*) that adopts the current EU case definition for this disease (*2*). Surveillance is mandatory and active throughout the year and enhanced during the season of vector activity when any suspected human case needs to be notified by medical doctors to the local health authorities (LHA) within 12 hours of detection. Clinical samples are sent to the Regional (where available) and/or National Reference Laboratories for Arboviruses for laboratory confirmation as previously described (*3*,*4*) according to a predefined diagnostic algorithm (*1*). Any probable or confirmed case notified to LHAs should be transmitted within 12 hours to the Italian Ministry of Health (MoH) and the National Institute of Health (ISS). LHAs also initiate environmental and epidemiologic investigations and alert vector control services to activate measures around each case (within 24 hours from notification) (*1*). Control measures on Substances of Human Origin (SoHO), are triggered when local transmission of multiple cases is ascertained.

Following the report of cases in the village of Guardavalle Marina, Calabria, clinically similar to those found in the Lazio region, an investigation was triggered to assess the size of the outbreak, explore epidemiologic links with other cases occurring in Italy, and set up effective control measures. The outbreak investigation team included clinicians, epidemiologists, medical entomologists and microbiologists from the Calabria Regional health system and from the ISS. The team investigated cases prospectively and retrospectively from the 26th of September 2017. Mosquito collection, identification and pooling (1–8 specimens) was performed as previously described (*5*) by the ISS. *Ae. albopictus* adults and larvae were collected within four selected sites and around the areas where the majority of cases had been reported as of 26–27 September

2017. The NRL in ISS, performed all the laboratory testing. Serology for IgM antibodies against CHIKV and plaque reduction neutralisation tests were performed as previously described (*6*). PCR was performed both on serum samples and on pools of *Ae. albopictus*. Sequencing of the amplicon in the E1 structural glycoprotein coding gene region was performed in all RT-PCR plus nested PCR positive samples (*7*), the phylogenetic analysis was based on available partial CHIKV E1 gene sequences. Tree reconstructions with MEGA version 4 used the neighbor-joining algorithm and the Kimura two-parameter distance model and reliability of analysis was confirmed through a bootstrap test with 1000 replications.

Statistical analysis included suspected, probable and confirmed human cases of CHIKV infection detected between August 2nd and October 30th 2017 with links to Guardavalle Marina. Dates of symptom onset were plotted in an epidemic curve and frequency distributions of the demographic and clinical characteristics of the patients were made. Attack rates (both overall and by age group and sex) were calculated for all probable and confirmed cases, with estimation of risk ratios (RR) and 95% CI. The spatio-temporal spread of the outbreak was analyzed from a subset of 81 cases geocoded in QGIS (version 2.8.14) (*8*) and mapped on OpenStreet Maps (*9*) in Guardavalle Marina. Significant case clusters were identified with the prospective space-time permutation model of SatScanTM (version 9.4) (*10*). Assuming cases and vector locations coincided, clusters were sought within the flight range of *Ae. albopictus* (maximum 200m) (*11*) within 10 days.

Between August 2nd and October 30th 2017, 132 Chikungunya cases (32 suspected, 25 probable, and 75 confirmed) were notified. Of those, 125 lived in Guardavalle Marina and the remaining had traveled there but presented symptoms in other Italian Regions (Emilia-Romagna, Lazio, and Lombardia). An 88 year-old suspected case with underlying medical conditions died, in all other cases the disease was self-limiting.

The epidemic curve, Figure 1, shows several epidemic waves. The peak of the outbreak occurred during the beginning of the fourth week of September, more than 6 weeks after the onset of symptoms on the first locally acquired case, and 7 weeks after the onset of symptoms in the presumed index case. This suggests that, at the peak, at least 3 transmission cycles had occurred (*12*). The earliest case detected was a suspected case who had traveled from Anzio to Guardavalle Marina on August 1st and developed fever and rash the following day.

Of the 100 confirmed and probable cases 53% were female. The mean age was 68 years (range 3–89). The clinically observed attack rate in Guardavalle Marina was 4.3% (100 cases among 2,346 inhabitants) and increased with age with the highest rates in people older than 60 years ($\chi 2$ for trend $p < 0.0001$). All probable/confirmed cases were febrile and 99% reported pain in multiple joints (Appendix Table 2).

We identified one significant cluster of six cases (p-value<0.05) (Figure 2a) occurring within a circle of 50m radius at the end of the epidemic (07/10/2017 to 16/10/2017). In the same area, three additional cases had occurred previously (from 22/09/2017 to 04/10/2017).

Thirty *Ae.albopictus* mosquitoes were collected and tested in 8 pools for CHIKV. CHIKV genome was detected in one pool of 8 mosquitoes and in 23 serum samples out of 75 confirmed. Sequences of the PCR amplicon of the virus envelope (E)1 gene were obtained from 22 serum samples and from the mosquito pool sample. Sequencing showed that the viral strain circulating in Guardavalle Marina (GenBank numbers: LT964945–67 and LT 964970) was very similar to the one circulating in Anzio in June-August 2017 (GenBank numbers: LT908477–78 and LT964968–69) (*13*), that had shown 100% similarity with the sequence of a Chikungunya ECSA strain circulating in early 2017 in Pakistan and since 2015 in India (*14*). None of these strains carried the A226V mutation. The phylogenetic analysis confirmed clustering of both Italian strains with other ECSA strain sequences (Figure 2b).

The epidemiologic and microbiological evidence collected during this investigation supported the hypothesis that the Guardavalle Marina outbreak originated from the Lazio Region rather than from an independent introduction. Measures for controlling *Ae. albopictus* density were implemented across Guardavalle Marina and around cases in other reporting regions using fast-acting insecticides (pyrethroid-based products) for 2 days consecutively, applied by a truck-mounted atomiser in public spaces and using a backpack mist blower in private spaces. Anti-larval measures, using formulations of insect growth regulators (I.G.R.) were also implemented. House-to-house interventions in Guardavalle Marina aimed to enhance community participation in identifying and eliminating/treating breeding sites. A second cycle of mosquito control interventions was carried out 1 week after the first one. Blood safety measures were implemented as previously described (*15*) from the 29/09/2017 (*16*) to 01/12/2017 (*17*).

References

1. Italian Ministry of Health. National Plan for the surveillance and response to arboviruses transmitted by *Aedes* sp. mosquitoes with specific reference to chikungunya, dengue and Zika – 2017 [in Italian] [cited 2019 Jan 8]. https://www.sanita.puglia.it/documents/36126/276493/Piano+Nazionale+di+sorveglianza+e+risposta+alle+arbovirosi+trasmesse+da+zanzare+%28Aedes+sp.%29+con+particolare+riferimento+ai+virus+Chikungunya%2C+Dengue+e+Zika+-+2017/a71cad75-033e-49b7-9469-208e07f5a144?version=1.0&t=1505722002455

2. The European Commission. Commission implementing decision (EU) 2018/945 of 22 June 2018. Decisions on the communicable diseases and related special health issues to be covered by epidemiological surveillance as well as relevant case definitions [cited 2019 Jul 31]. https://eur-lex.europa.eu/legal-content/EN/TXT/PDF/?uri=CELEX:32018D0945&from=EN#page=13

3. Rezza G, Nicoletti L, Angelini R, Romi R, Finarelli AC, Panning M, et al.; CHIKV Study Group. Infection with chikungunya virus in Italy: an outbreak in a temperate region. Lancet. 2007;370:1840–6. PubMed https://doi.org/10.1016/S0140-6736(07)61779-6

4. Edwards CJ, Welch SR, Chamberlain J, Hewson R, Tolley H, Cane PA, et al. Molecular diagnosis and analysis of chikungunya virus [cited 2019 Jan 8]. https://www.sciencedirect.com/science/article/pii/S1386653207001850?via%3Dihub

5. Venturi G, Di Luca M, Fortuna C, Remoli ME, Riccardo F, Severini F, et al. Detection of a chikungunya outbreak in central Italy, August to September 2017. Euro Surveill. 2017;22(39):pii=17–00646. https://doi.org/10.2807/1560-7917.ES.2017.22.39.17-00646

6. Fortuna C, Remoli ME, Rizzo C, Benedetti E, Fiorentini C, Bella A, et al.; Arbovirus Working Group. Imported arboviral infections in Italy, July 2014–October 2015: a National Reference Laboratory report. BMC Infect Dis. 2017;17:216. doi:10.1186/s12879-017-2320-1

7. Rezza G, Nicoletti L, Angelini R, Romi R, Finarelli AC, Panning M, et al.; CHIKV Study Group. Infection with chikungunya virus in Italy: an outbreak in a temperate region. Lancet. 2007;370:1840–6. PubMed https://doi.org/10.1016/S0140-6736(07)61779-6

8. QGIS Development Team. QGIS Geographic Information System. Open Source Geospatial Foundation Project [cited 2018 Mar 30]. http://qgis.osgeo.org

9. OpenStreetMap contributors. Planet dump [cited 2018 Mar 30]. https://planet.osm.org

10. Kulldorff M, Heffernan R, Hartman J, Assunção RM, Mostashari F. A space-time permutation scan statistic for the early detection of disease outbreaks. PLoS Med. 2005;2:216–24.

11. European Centre for Disease Prevention and Control. *Aedes albopictus*–Factsheet for experts. [cited 2017 Dec 21]. http://ecdc.europa.eu/en/disease-vectors/facts/mosquito-factsheets/aedes-albopictus

12. Pan American Health Organization/World Health Organization. Chikungunya: a new virus in the region of the Americas [cited 2018 Mar 30]. http://www.paho.org/hq/index.php?option=com_content&view=article&id=9724%3A2014-chikungunya-un-nuevo-virus-en-la-region-de-las-americas&catid=1443%3Aweb-bulletins&Itemid=135&lang=en

13. Bordi L, Carletti F, Lalle E, Colavita F, Meschi S, Di Caro A, et al. Molecular characterization of autochthonous chikungunya cluster in Latium Region, Italy. Emerg Infect Dis. 2018;24:178–80. https://dx.doi.org/10.3201/eid2401.171605

14. Lindh E, Argentini C, Remoli ME, Fortuna C, Faggioni G, Benedetti E, et al. The Italian 2017 outbreak chikungunya virus belongs to an emerging *Aedes albopictus* adapted virus cluster introduced from the Indian subcontinent [cited 2019 Jan 7]. https://academic.oup.com/ofid/advance-article/doi/10.1093/ofid/ofy321/5239584

15. Liumbruno GM, Calteri D, Petropulacos K, Mattivi A, Po C, Macini P, et al. The chikungunya epidemic in Italy and its repercussion on the blood system. Blood Transfus. 2008;6:199–210. PubMed

16. Guardavalle Online. Measures to suspend (blood donations) due to chikungunya virus (CHIKV) in the municipality of Guardavalle [in Italian] [cited 2019 Aug 28]. https://www.guardavalle.online/misure-sospensione-chikungunya-virus-chikv-nel-comune-guardavalle

17. Italian National Blood Centre. Directive for the discontinuation (of blood safety restrictions) aimed at preventing the transmission of Chikungunya virus infections [in Italian] [cited 2018 Mar 30]. http://www.centronazionalesangue.it/sites/default/files/Prot.%20n.%202946.CNS_.2017_Sospensione%20misure%20di%20prevenzione%20della%20trasmissione%20dell%27infezione%20da%20virus%20Chikungunya%20-%20Comuni%20di%20Anzio%20%28Roma%29%20e%20Guardavalle%20Marina%20%28CZ%29.pdf#overlay-context=node/573

Appendix Table 1. Demographic characteristics of confirmed and probable Chikungunya cases (n = 100), clinically observed attack rates and Risk Ratio, Guardavalle Marina (CZ), August – October 2017

Age (years)	Number of Chikungunya cases (n = 100)	Attack Rate	Risk Ratio (IC 95%)
0–19	5	1.0%	Ref.
20–39	8	1.2%	
40–59	24	3.8%	3.32 (1.70 – 6.47)
60–79	53	13.5%	11.87 (6.54 - 21.53)
≥80	10	5.6%	4.93 (2.20 - 11.07)
Sex			
Male	47	4.1%	Ref.
Female	53	4.4%	1.09 (0.74 - 1.60)

Appendix Table 2. Frequency of reported symptoms among confirmed and probable Chikungunya cases (n = 100), Guardavalle Marina (CZ), August – October 2017

Symptom	Number of cases (%)
Fever	100 (100)
Joint pain	99 (99)
Rash	52 (52)
Headache	40 (40)
Fatigue	38 (38)
Muscle pain	36 (36)
Diarrhea	6 (6)
Itching	6 (6)
Vomit	5 (5)

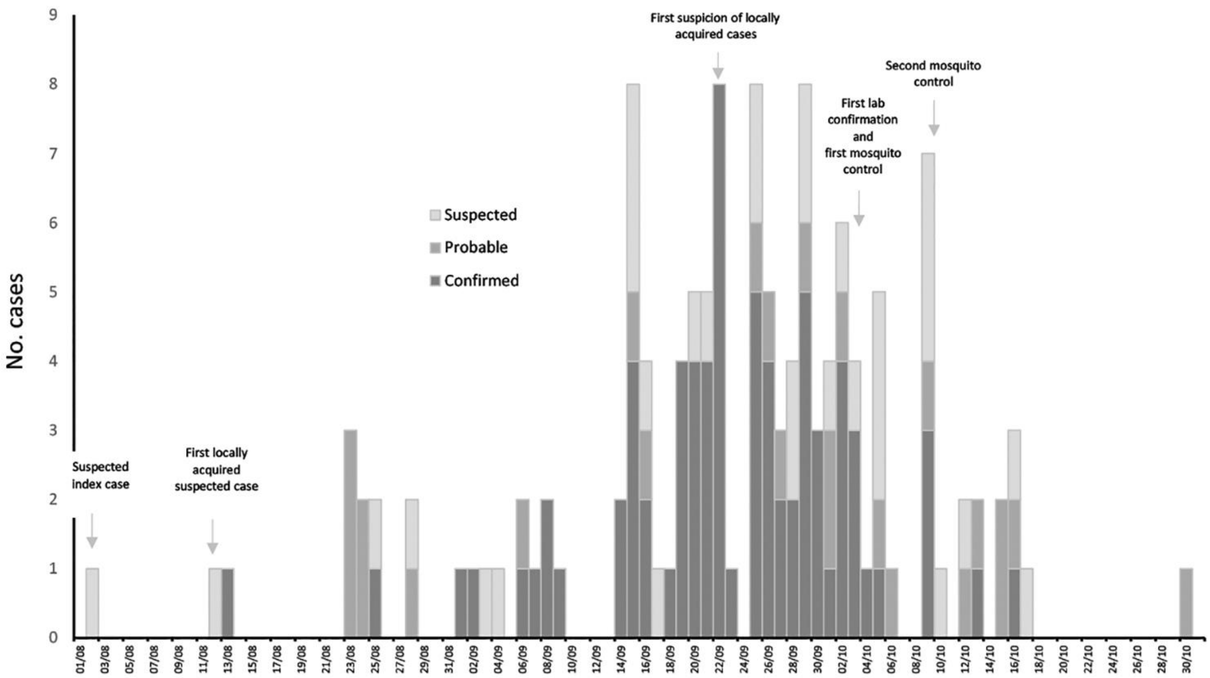

Appendix Figure 1. Epidemic curve of suspected, probable and confirmed Chikungunya cases (n = 132) occurring in, or with travel history to, Guardavalle Marina (CZ), timeline of outbreak detection and vector control activities, August – October 2017.

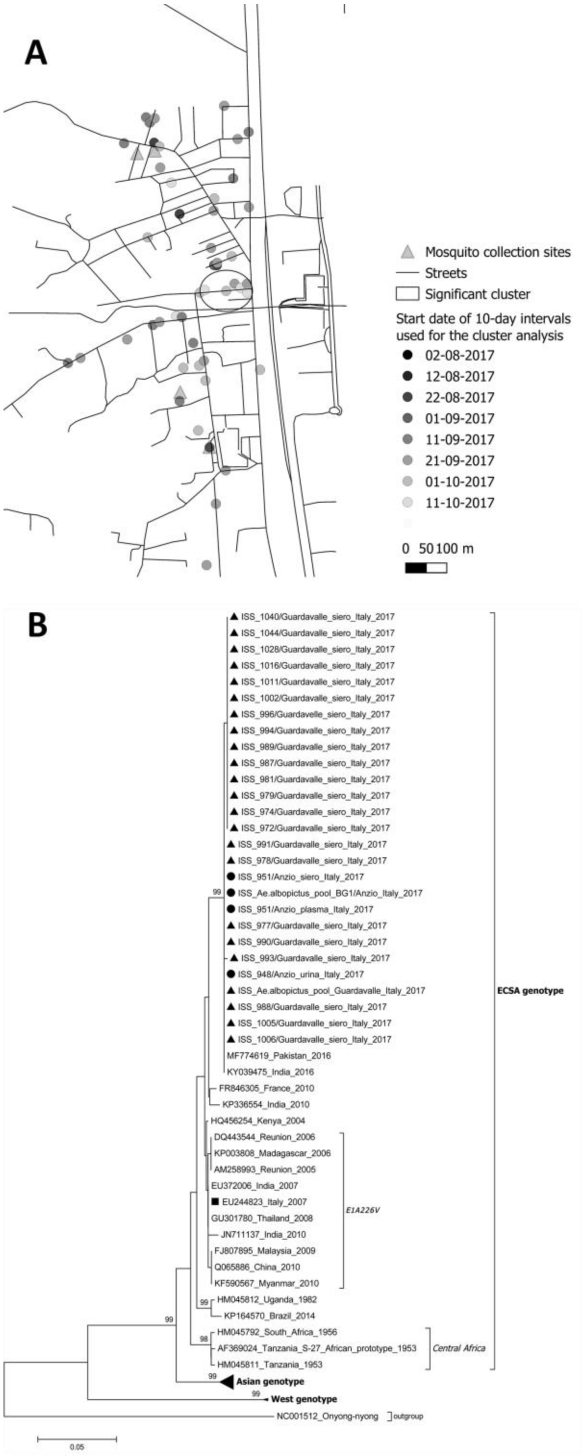

Appendix Figure 2. A) Cases in Guardavalle Marina, Italy, 2017, gray-scale coded by 10-day intervals with clustering analyses (circles) and mosquito collection sites (triangles). B) Neighbor-joining phylogenetic analysis of sequences derived from 22 chikungunya virus–positive samples from patients and from 1 pool of mosquitoes from Guardavalle Marina, Italy, August-October 2017.
